# Prognostic value of oxidative phosphorylation-related genes in hepatocellular carcinoma

**DOI:** 10.1007/s12672-024-01129-3

**Published:** 2024-07-03

**Authors:** Luzheng Liu, Jiacheng Chen, Fei Ye, Fengran Chu, Chaoluan Rao, Yong Wang, Yanggang Yan, Jincai Wu

**Affiliations:** 1 Department of Interventional Radiology and Vascular Surgery, The Second Affiliated Hospital of Hainan Medical University, Haikou, 570100 Hainan Province China; 2 Department of Hepatobiliary and Pancreatic Surgery, Hainan General Hospital, Hainan Affiliated Hospital of Hainan Medical University, Haikou, 570311 Hainan Province China; 3 Department of Blood Cell Therapy, The Second Affiliated Hospital of Hainan Medical University, Haikou, 570100 Hainan Province China; 4 Department of Nursing, The Second Affiliated Hospital of Hainan Medical University, Haikou, 570100 Hainan Province China

**Keywords:** HCC, Oxidative phosphorylation genes, Prognosis, Immunotherapy, Nomogram, Real time quantitative PCR

## Abstract

**Purpose:**

Hepatocellular carcinoma (HCC) is the most prevalent malignancies worldwide. Recently, oxidative phosphorylation (OXPHOS) has received extensive concern as an emerging target in antitumor therapy. However, the OXPHOS-involved underlying genes and clinical utilization in HCC remain worth exploring. The present research aimed to create an OXPHOS-relevant signature in HCC.

**Patients and methods:**

In this study, the prognostic signature genes linked with OXPHOS were identified, and prognostic models were built using least absolute shrinkage and selection operator (LASSO) cox regression analysis. Furthermore, the combination study of immune microenvironment and signature genes looked into the involvement of immune cells in signature-based genes in HCC. Following that, chemotherapeutic drug sensitivity and immunotherapy analysis was implemented to predict clinical efficacy in HCC patients. Finally, clinical samples were collected to measure the expression of OXPHOS-related signature genes.

**Results:**

Following a series of screens, six prognostic signature genes related with OXPHOS were identified: MRPS23, MPV17, MAPK3, IGF2BP2, CDK5, and IDH2, on which a risk model was built. The findings revealed a significant drop in the survival rate of HCC patients as their risk score increased. Meanwhile, independent prognostic study demonstrated that the risk score could accurately identify HCC patients. Immuno-microenvironmental correlation research suggested that the prognostic characteristics could serve as a reference index for both immunotherapy and chemotherapy. Finally, RT-qPCR exhibited a trend in signature gene expression that was consistent with the results.

**Conclusion:**

In this study, a total of six prognostic genes associated with OXPHOS were selected and a prognostic model was constructed, providing an essential reference for the study of OXPHOS in HCC.

## Introduction

Liver cancer (LC) is a common, malignant, heterogeneous tumor that ranked sixth in incidence and third in mortality among cancers worldwide [1]. As the most frequent pathology, the occurrence of hepatocellular carcinoma (HCC) is associated with a variety of factors such as viral infection and cirrhosis of the liver [2]. Currently, the main effective treatments for HCC are hepatectomy, transhepatic arterial chemotherapy and embolization, and liver transplantation [3, 4]. However, even with advances in treatment management, overall outcomes for HCC patient survival are not ideal with low 5-year survival rates and high rates of metastatic recurrence, posing a greater challenge to clinical caregivers [5]. Therefore, it is of significance to explore more specific HCC markers and models to provide appropriate treatment for patients and improve their prognosis of patients.

Recently, abnormalities in cancer cell metabolism have supported metabolic plasticity and high energy production during cell migration and metastasis [6, 7]. It was found that interferon-stimulated gene 15 (ISG15), in more detail, the ISGylation mechanism ensures the circulation of dysfunctional mitochondria that drive tumourigenesis, chemoresistance, and metastasis in pancreatic cancer, whereas ISGylation plays a crucial role for an optimized and efficient oxidative phosphorylation (OXPHOS) [8]. OXPHOS is an emerging target for cancer development and therapy [9]. New evidence of triple-negative breast cancer suggests that inhibition of OXPHOS may magnify amplify the superiority of multiple anti-tumor therapies [10, 11]. With transmitting the bioenergy and controlling the macromolecular anabolic process, there is a molecular and therapeutic implication for OXPHOS pathway in tumors [12]. For the clinical utility of diagnostic markers, it has been more reliable through the use of various analytical methods to construct prognostic and diagnostic models [13, 14]. It is noteworthy that the OXPHOS-related prognostic performance for HCC patients has not yet been investigated.

In this research, the bioinformatics approach was applied to establish an OXPHOS-related risk signature for predicting survival in HCC patients, aiming to provide a theoretical foundation for the exploration of advanced treatment strategies for HCC.

## Material and methods

### Gene and datasets collection

We integrated transcriptomic data, somatic mutation data, and associated clinical data of 371 HCC tissue specimens and 50 normal specimens through the Cancer Genome Atlas (TCGA) database (https://portal.gdc.cancer.gov/). GSE74627 (115 HCC samples) and GSE50579 (67 HCC samples and 10 controls) were downloaded from the Gene Expression Omnibus (GEO) database (https://www.ncbi.nlm.nih.gov/geo/). In addition, the transcriptomic data and survival time records of 343 HCC tissue specimens were acquired from International Cancer Genome Consortium (ICGC) database (https://docs.icgc.org/). ICGC-HCC dataset and GSE74627 dataset were incorporated into prognostic model verification. 230 OXPHOS genes were derived from NCBI (keyword ‘OXPHOS’) and Metabolic Atlas (keyword ‘Oxidative phosphorylation’) databases after de-duplication.

### Variance expression analysis

On the basis of P < 0.05 and |log_2_FoldChange (FC)|> 0.5, the differentially expressed genes (DEGs) were determined with ‘edge R’ package (version 3.34.1) [15]. Moreover, Gene Ontology (GO) and Kyoto Encyclopedia of Genes and Genomes (KEGG) enrichment analyses were utilized via the ‘clusterProfiler’ (version 4.0.2) [16], where GO was segmented into different cellular component (CC), molecular function (MF), and biological process (BP) with p value < 0.05.

### Establishment of OXPHOS-relevant signature in HCC

Firstly, 350 HCC patients with complete survival data in TCGA-HCC cohort were used to construct the signature as training set. The univariate Cox (‘survival’ package (version 3.2-13)) and Least absolute shrinkage and selection operator (LASSO) regression (‘glmnet’ package (version 4.1-3)) analysis were deployed to select survival-associated OXPHOS genes [17]. According to the median of OXPHOS-relevant risk score, patients were separated into high risk and low risk subgroups. ICGC-HCC dataset (343 HCC samples), and GSE76427 (115 HCC samples) datasets were served as external validation sets. Kaplan–Meier (K–M) curves, receiver operating characteristic (ROC) analysis, and risk curves were generated for assessment prognosis of the signature obtained.

### Exploration of independent prognostic predictors and nomogram

The OXPHOS-relevant signature and clinical pathological factors were enrolled into Cox analysis (univariate Cox and multivariate Cox) to authenticate whether had independent prognostic value. Using the prognostic factors selected with P < 0.05, nomogram and calibration curves were pictured by ‘rms’ (version 6.2-0) and ‘regplot’ (version 1.1) for survival prediction of HCC patients [18].

### Enrichment analysis for the OXPHOS-relevant signature

The Gene Set Enrichment Analysis (GSEA) was further implemented for biological significance of the signature genes [19]. Specifically, the terms accorded with the |normalized enrichment scores (NES)|greater than 1, NOM p > 0.05 as well as q < 0.25 were set as significantly enriched.

Inference of tumor microenvironment (TME) based on the OXPHOS-relevant risk model Immune score, stromal score and ESTIMATE score were calculated for tumor purity resolution via ESTIMATE algorithm [20].The discrepancies in 24 immune cells abundance for different risk populations were assessed by the ssGSEA algorithm (‘GSVA’ package (version 1.40.1)) and wilcoxon test [21].

### Mutation and therapy analysis based on the OXPHOS-relevant risk model

To investigate the relevance of OXPHOS-relevant risk signature to somatic mutations, we conducted mutation analysis using the ‘maftools’ package (version 2.8.05) [22].The response to immune checkpoint blockade (ICB) therapy for different populations was compared by the Tumor Immune Dysfunction and Exclusion (TIDE) website [23]. Via the ‘oncoPredict’ package (version 0.2) [24], we computed half-limiting dose (IC50) values for 198 chemotherapy drugs for two risk subgroups of patients to speculate on their sensitivity to chemotherapy.

### Mutation analysis and establishment of a transcription factor (TF) regulation network based on OXPHOS-relevant prognostic genes

Genetic alterations in OXPHOS-relevant prognostic genes were profiled on GSCALite database (http://bioinfo.life.hust.edu.cn/web/GSCALite/). We projected TFs regulating prognostic genes utilizing the Cistrome database (Filtering criteria: RP score > 0.4) and the NetworkAnalyst online visual analysis platform. The intersecting TFs of TFs obtained above and DEGs between HCC and normal tissue specimens were incorporated into a TF-prognostic gene regulatory network created by Cytoscape software.

### Validation for the signature genes expression using the real time quantitative PCR (RT-qPCR)

The discrepant expression of the prognostic genes involved between HCC samples and controls in GSE50579 were firstly examined. Furthermore, eight paired HCC and paracancerous tissue samples were extracted from patients of Hainan Affiliated Hospital of Hainan Medical University with the approval of the ethics committee as well as informed consent. After total RNA extracting through the TRIzol (Ambion, Austin, USA). The inverse transcription procedure was conducted under the producer’s indication. Then, the 2xUniversal Blue SYBR Green qPCR Master Mix was further adopted for qPCR operation (Servicebio, Wuhan, China). The primer sequences involved were tabulated as follow (F: Forward; R: Reverse):

MRPS23 F: 5′-CTCGGACTCGGGACCTGGTT-3′.

MRPS23 R: 5′-GATGGGAGCTTTGGCTTTGC-3′.

MPV17 F: 5′-GCTCACCCGTGGAAAGTACAG-3′.

MPV17 R: 5′-GTGGGAGAAAGCAGCCTAGAA-3′.

MAPK3 F: 5′-ACATCCACTCCGCCAACG-3′.

MAPK3 R: 5′-TCAGCCAGAATGCAGCCC-3′.

IGF2BP2 F: 5′-CACTACCACGTTGATGGCTTTC-3′.

IGF2BP2 R: 5′-GTTCCACATTCTCCACTGTCCC-3′.

CDK5 F: 5′-CCTGCACAGCGACAAGAAGC-3′.

CDK5 R: 5′-GTAACAGCGGACGGGAATCC-3′.

IDH2 F: 5′-CCGTCACCCGCCACTATC-3′.

IDH2 R: 5′-CCGTCTCCACGCACACCT-3′.

GAPDH F: 5′-ACAACTTTGGTATCGTGGAAGG-3′.

GAPDH R: 5′-GCCATCACGCCACAGTTTC-3′. The gene expression was computed employing the 2^−ΔΔCt^ method [25].

### Statistical analysis

All bioinformatics analyses were undertaken in R language. Wilcoxon test and Kruskal–Wallis test were employed for differences in different groups, and Student’s t-test was utilized for discrepancies in RT-qPCR. If not otherwise stated, P < 0.05 represents the significance.

## Results

### The OXPHOS-relevant gene signature for assessing the prognosis of HCC patients

**Fig. 1:**
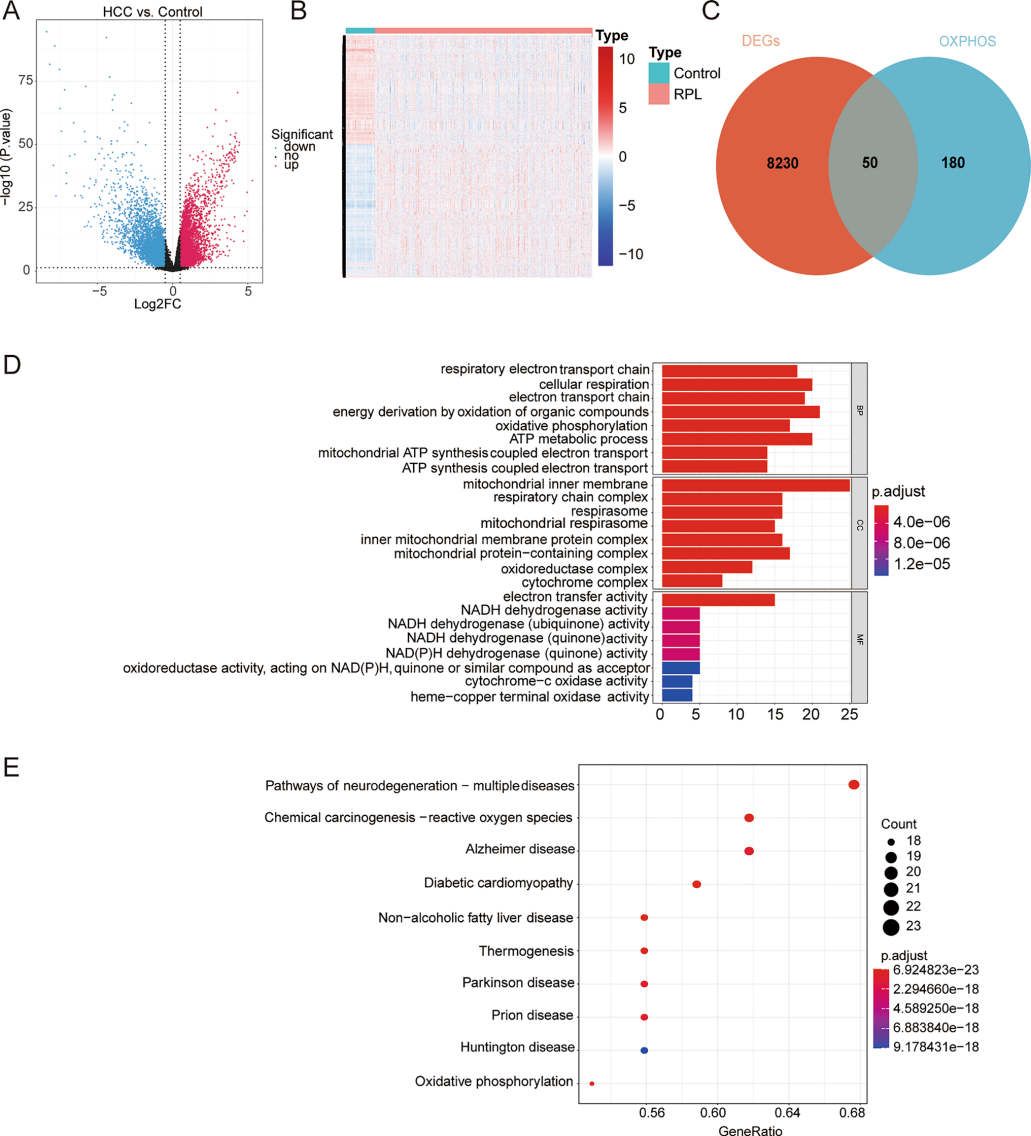
The OXPHOS-relevant gene signature for assessing the prognosis of HCC patients. The 50 DE-OXPHOS genes were mainly linked to ‘Chemical carcinogenesis—reactive oxygen species’, ‘Oxidative phosphorylation’. **A** Volcano map of differentially expressed genes (DEGs) between HCC and normal samples from the TCGA database. **B** Heatmap of DEGs. **C** Venn diagram of oxidative phosphorylation (OXPHOS) genes and DEGs. **D** Gene Ontology (GO) enrichment analysis of DE-OXPHOS genes was conducted based on the GO database, and the bar chart exhibiting the Top8 items under cellular component (CC), molecular function (MF), and biological process (BP). **E** The DE-OXPHOS genes were mapped into the Kyoto Encyclopedia of Genes and Genomes (KEGG) database for pathway analyses, and the bubble chart exhibiting the Top10 KEGG pathways

On the basis of the 8280 DEGs in two groups of TCGA-HCC cohort (4380 genes were highly expressed and 3900 genes were lowly expressed in HCC) (Fig. 1A, B) and 230 OXPHOS common to NCBI and the Metabolic Atlas databases, we obtained 50 DE-OXPHOS genes (Fig. 1C, Table 1). We observed that these DE-OXPHOS genes were mainly linked to ‘Chemical carcinogenesis—reactive oxygen species’, ‘Oxidative phosphorylation’ (Fig. 1D, E).

**Table 1:** The list of differentially expressed oxidative phosphorylation (DE-OXPHOS) genes

DE-OXPHOS genes
ANGPTL6
COX4I2
ETFDH
ACAD11
MRPS23
SDHD
CDK5
MPV17
SDHB
MAPK3
SNHG3
SDHA
CKS2
NES
PARP1
TOMM20
ETFA
NDUFS1
MIPEP
PPIF
TIMM50
COX6A2
UQCRC2
MFN2
PRKAA1
MYC
ATP5J2
COX7B2
PPARGC1A
IDH2
NDUFV2
SUCLA2
EIF5A
LHPP
SQSTM1
NFKB1
UQCRH
SLC16A1
CYC1
NDUFA1
NDUFS6
UQCRB
COX6C
NDUFB9
SPINK1
PLAUR
SRC
UCP2
IGF2BP2
COX6B2

**Fig. 2:**
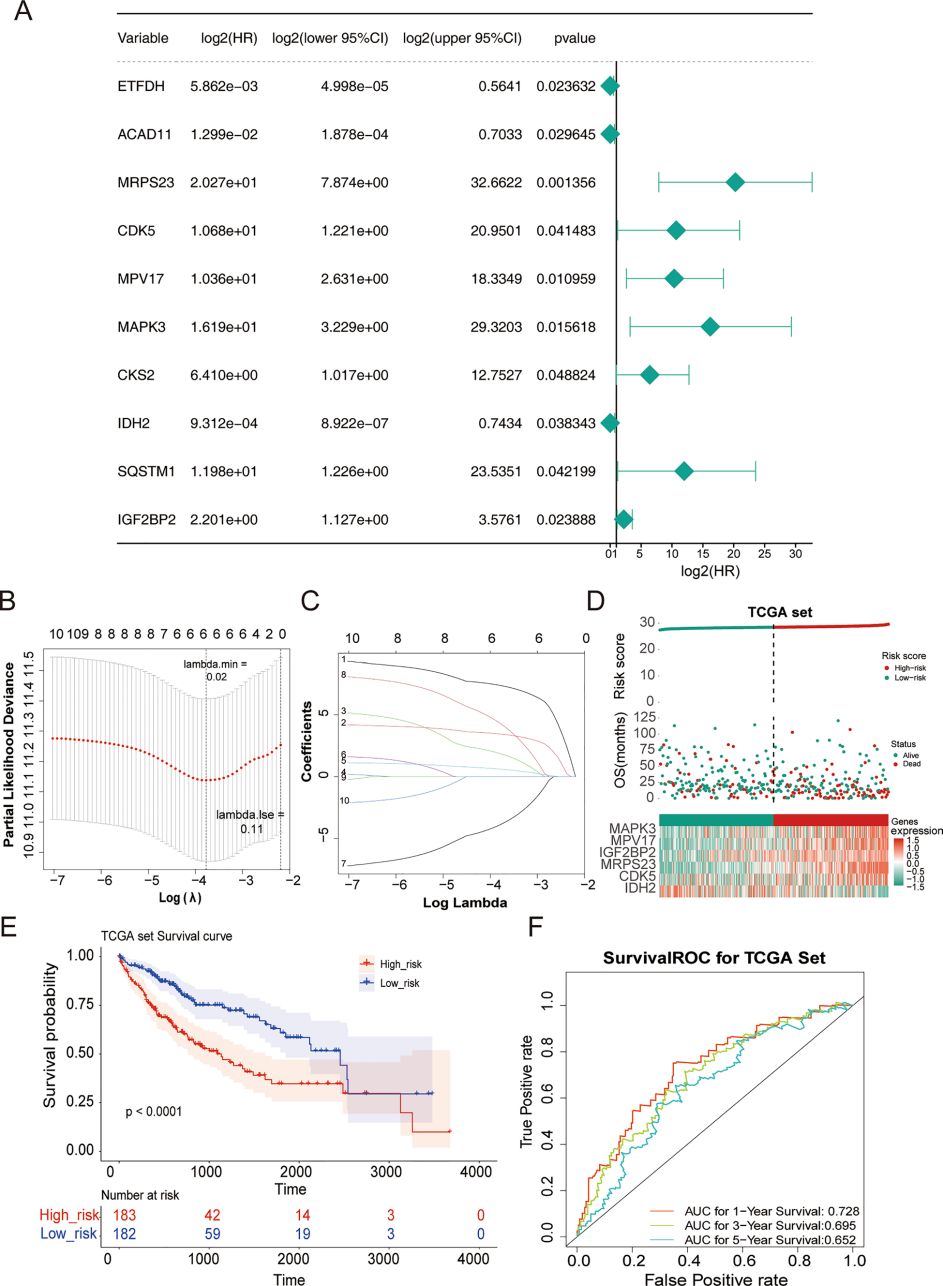
The OXPHOS-relevant gene signature for assessing the prognosis of HCC patients. There was a substantial difference in survival between the high and low risk groups.** A** Forest plot of univariate Cox regression analysis. Hazard ratio (HR) was the risk ratio, lower 95%CI, and upper 95%CI are the 95% confidence intervals of the risk value. **B** The Least absolute shrinkage and selection operator (LASSO) coefficient diagram of 10 genes in HCC. **C** Selecting the best parameters for HCC based on the LASSO model (λ). **D** Risk score distributions, patient survival status, and gene expression profiles in TCGA-HCC. **E** Kaplan–Meier (K–M) analysis between the high-risk group and low-risk group in TCGA-HCC. **F** Receiver operator curve (ROC) for the OXPHOS-relevant risk signature in TCGA-HCC

**Fig. 3:**
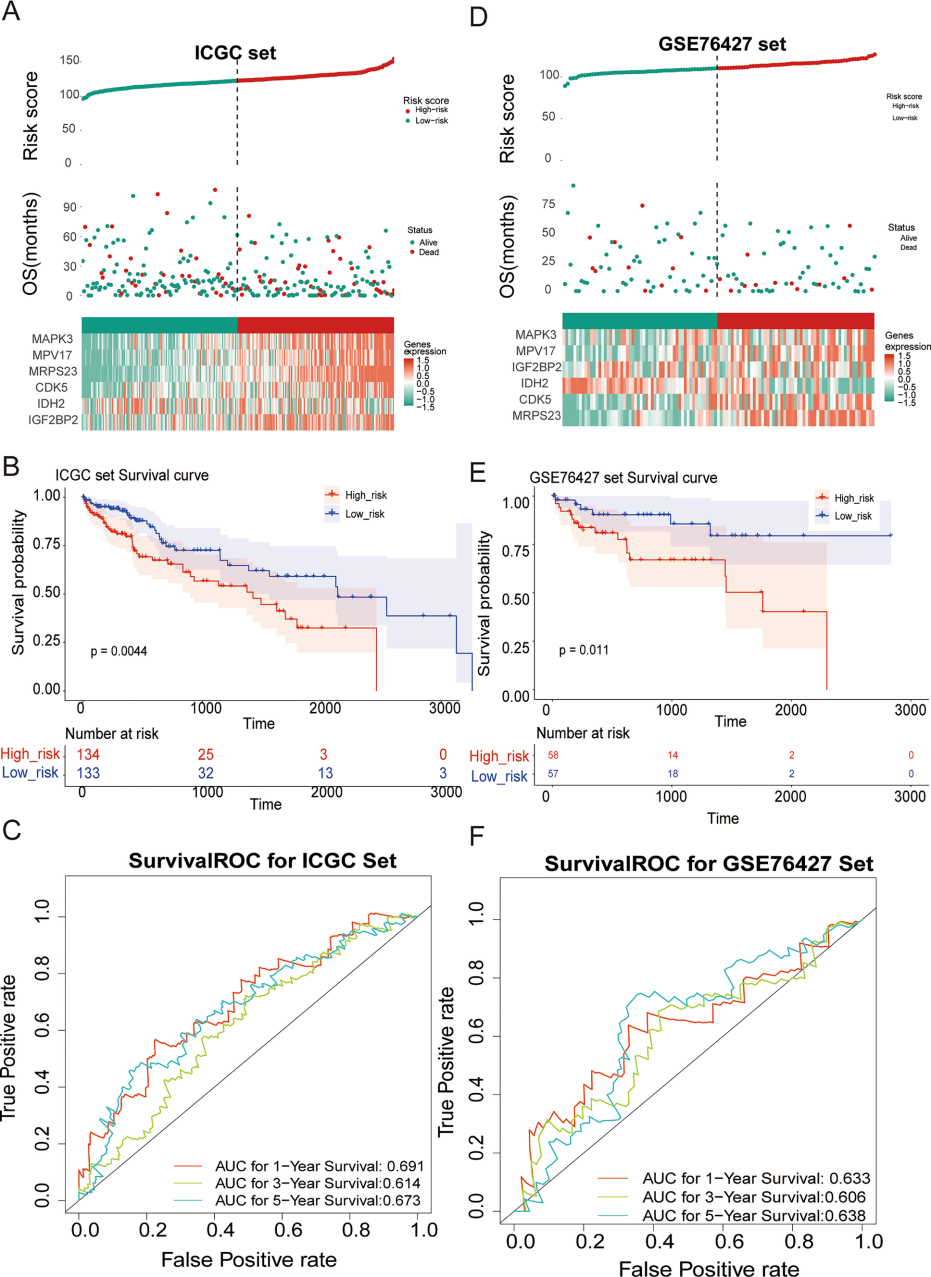
The OXPHOS-relevant gene signature for assessing the prognosis of HCC patients. The ICGC-HCC and GSE74627 datasets verified the accuracy of predicting survival between high- and low-risk groups separated by risk score. **A** Risk score distributions, patient survival status, and gene expression profiles in the external validation set of ICGC-HCC. The vertical reference line represents the sample position corresponding to the median risk score value, with low-risk patients on the left and high-risk patients on the right of the reference line. **B** K–M curve between the high-risk group and low-risk group of ICGC-HCC. **C** The ROC analysis for the OXPHOS-relevant risk signature in ICGC-HCC. **D** Risk score distributions, patient survival status, and gene expression profiles in the external validation set of GSE74627. **E** K–M curve analysis between the high-risk group and low-risk group of GSE74627. **F** ROC analysis for the OXPHOS-relevant risk signature in GSE74627

Using the univariate Cox (Fig. 2A) and LASSO analysis (Fig. 2B, C), six survival-related genes (MRPS23, MPV17, MAPK3, IGF2BP2, CDK5, and IDH2) were determined as optimal OXPHOS-relevant genes for HCC prognosis. Next, an OXPHOS-relevant risk signature was generated, at meantime, HCC populations in the training set were classified into two distinct risk subgroups (high risk and low risk) relying on the median risk scores (Fig. 2D). Especially, except for IDH2, other prognostic genes expressed higher in high-risk groups. Besides, significantly different survival probabilities were observed between the two groups from KM curves (Fig. 2E). The ROC curves for the signature were graphed and among the AUC values in the training set were greater than 0.6 at 1-, 3-, and 5- years (Fig. 2F). Likewise, similar analytical methods involved were adopted in ICGC-HCC (Fig. 3A–C) and GSE74627 (Fig. 3D–F) datasets, confirming the stable predictive accuracy of the signature.

### Construction and evaluation of nomogram

**Fig. 4:**
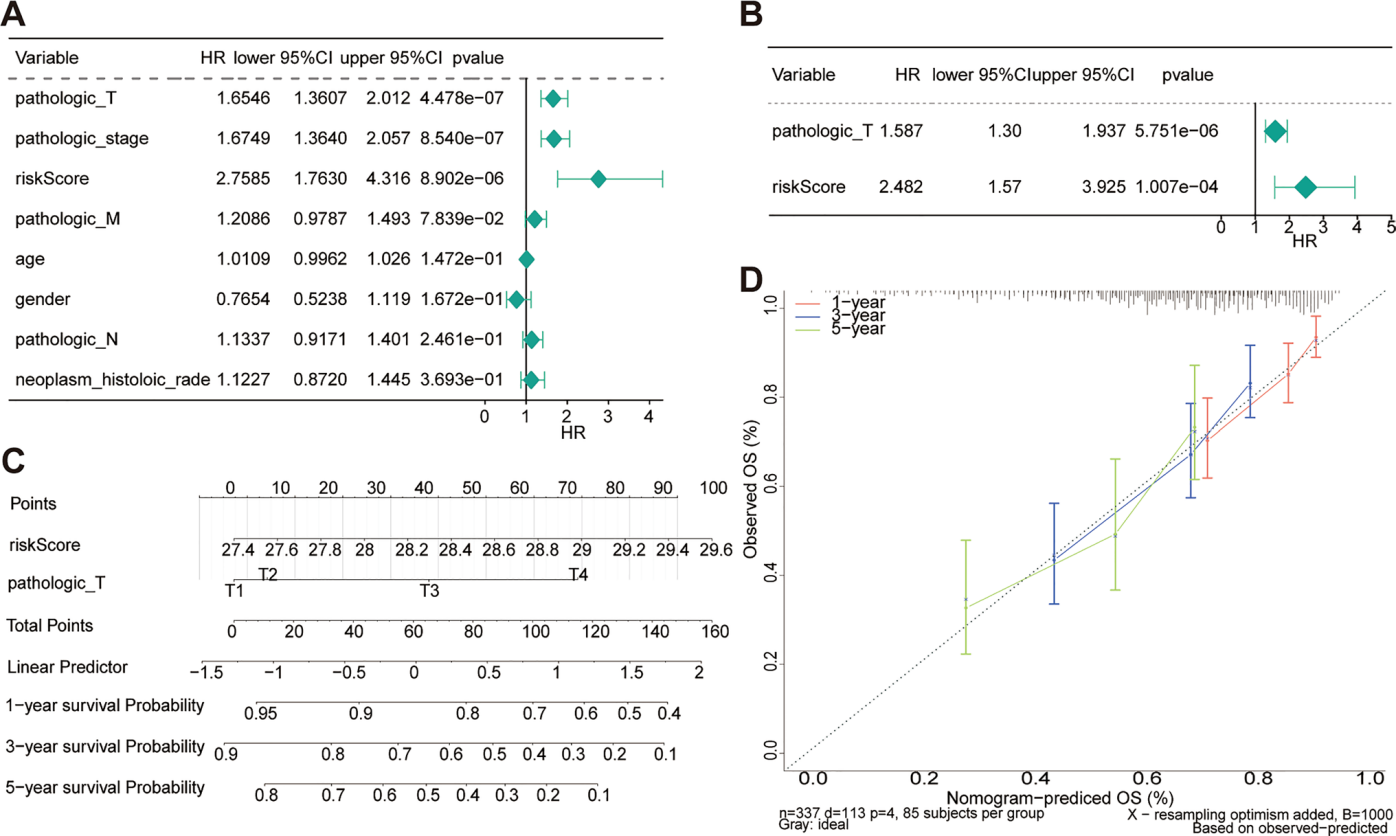
Construction and evaluation of nomogram. Validation of the independence of the OXPHOS-relevant risk signature. Forest plots for the (**A**) univariate cox regression analysis and (**B**) multivariate cox regression analysis. **C** A nomogram was constructed to predict the prognosis of HCC in TCGA-HCC. **D** The calibration plots at 1, 3, and 5-years for the nomogram predicting the prognosis of HCC in TCGA-HCC

Via the univariate Cox and multivariate Cox analysis, the signature model and pathologic T were found as independent predictive factors for HCC (Fig. 4A, B, P < 0.05). The C-index of nomogram associated reached 0.6621 and the calibration curves were used to further determine the valid capability of nomogram (Fig. 4C, D).

### The potential biological significances and tumor microenvironment (TME) differences of the different risk groups

**Fig. 5:**
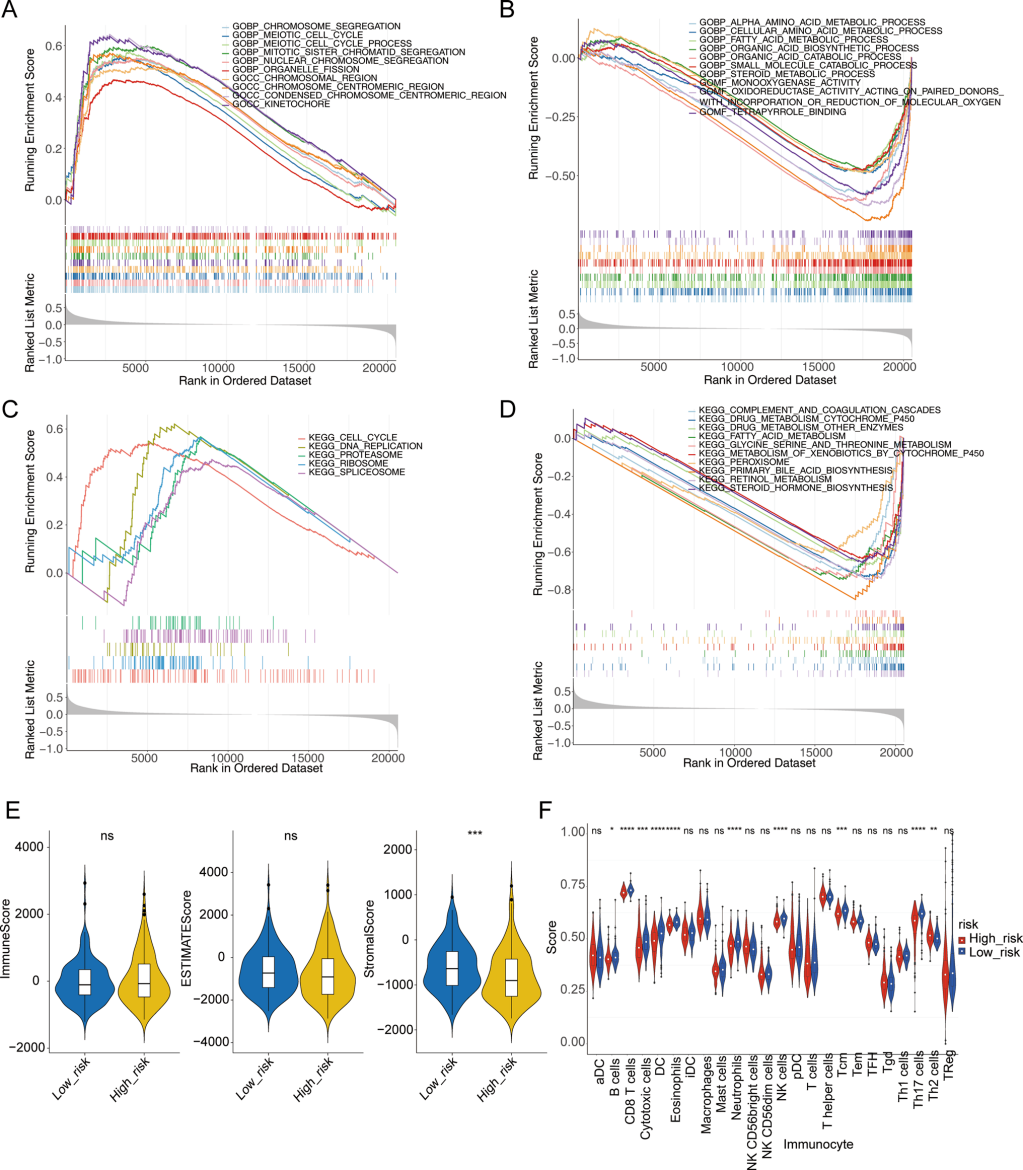
The potential biological significances and tumor microenvironment (TME) differences of the different risk groups. In the high-risk group, the stromal and Th2 cell scores were higher. **A** Top10 GO items for high-risk group. **B** Top10 GO items for low-risk group. **C** KEGG pathways enriched for high-risk group. **D** Top10 KEGG pathways enriched for low-risk group. For **A–D**, the enrichment maps were generated according to the GSEA database. **E** The ESTIMATE score, immune score, and stromal score in high- and low-risk subgroups. **F** Comparison of the scores of tumor-infiltrating immune cells between different risk subgroups. ^a^P < 0.05, ^b^P < 0.01, ^c^P < 0.001, ^d^P < 0.0001

GSEA was further conducted in the two OXPHOS-relevant risk subgroups for function enrichment analysis. Obviously, multiple cell cycle-related biological processes and pathways were activated in the patients with higher OXPHOS-relevant risk scores (NES > 0). Metabolism-related biological processes and pathways, calcium signaling pathway, PPAR signaling pathway, and complement and coagulation cascades were activated in the populations whose had lower OXPHOS-relevant risk scores (NES < 0) (Fig. 5A–D). This evidence suggested that the OXPHOS-relevant gene signature might predominantly act on the cell cycle and metabolism-related pathways in HCC progression.

On the other hand, it was demonstrated that only stromal score was remarkably higher in high-risk populations (Fig. 5E). And meanwhile, the score of Th2 cells was elevated in high-risk populations, on the contrary, there were 9 immune cells expressed higher in low-risk populations, such as B cells, CD8 T cells, DC, Eosinophils (Fig. 5F).

### Association of OXPHOS-relevant gene signature with therapy

**Fig. 6:**
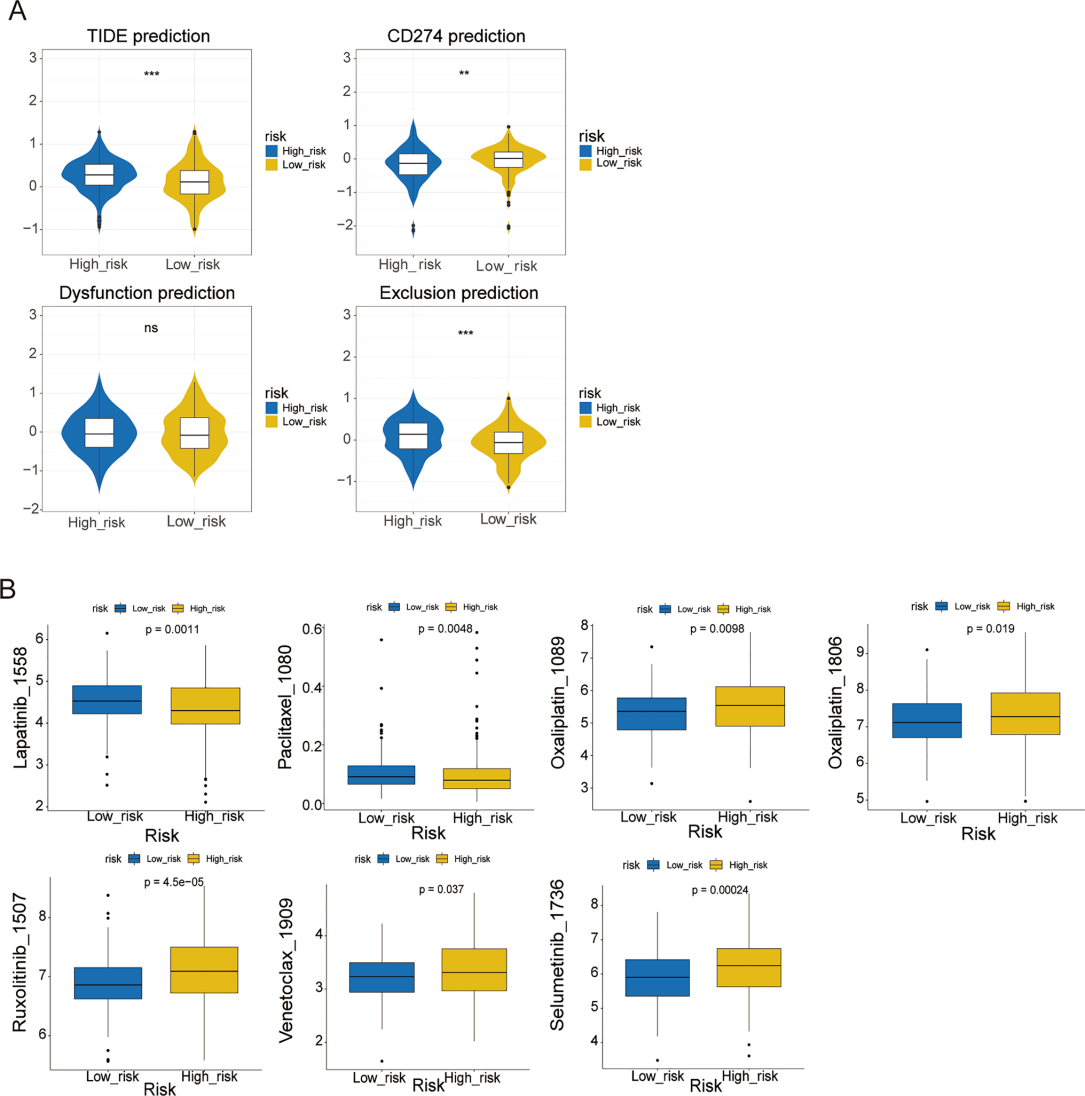
Association of OXPHOS-relevant gene signature with therapy. Patients with lower risk scores respond better to immunotherapy than patients with higher risk scores. **A** Comparative analyses of susceptibility to immune checkpoints and immunotherapy in high- and low-risk subgroups, including TIDE prediction, CD274 prediction, Dysfunction prediction, and Exclusion prediction. **B** The chemotherapy response of the different risk subgroups was predicted based on the public pharmacogenomic database. ^a^P < 0.05, ^b^P < 0.01, ^c^P < 0.001, ^d^P < 0.0001

For the TIDE results, it was presented that the populations with lower risk scores had inferior TIDE score and T cell exclusion score, implying better adaptability for immunotherapy in low risk populations compared with patients with higher risk scores (Fig. 6A). The anti-PD-L1 therapy score of patients with lower risk scores were notably superior than patients with higher risk scores. Next, we investigated whether the OXPHOS-relevant gene signature could predict sensitivity to chemotherapy drugs. Through the oncoPredict package, we calculated the IC50 values for each sample in two OXPHOS-relevant risk subgroups. We noticed that the OXPHOS-relevant high-risk group was more sensitive to lapatinib and paclitaxel, and the populations with low OXPHOS-relevant risk scores were more sensitive to oxaliplatin, ruxolitinib, selumetinib, venetoclax and multiple antineoplastic drugs (Fig. 6B). These findings suggested that the OXPHOS-relevant signature may be able to act as a chemosensitivity predictor for the choice of drugs for clinical treatment.

### Mutation analysis and TF regulatory network

**Fig. 7:**
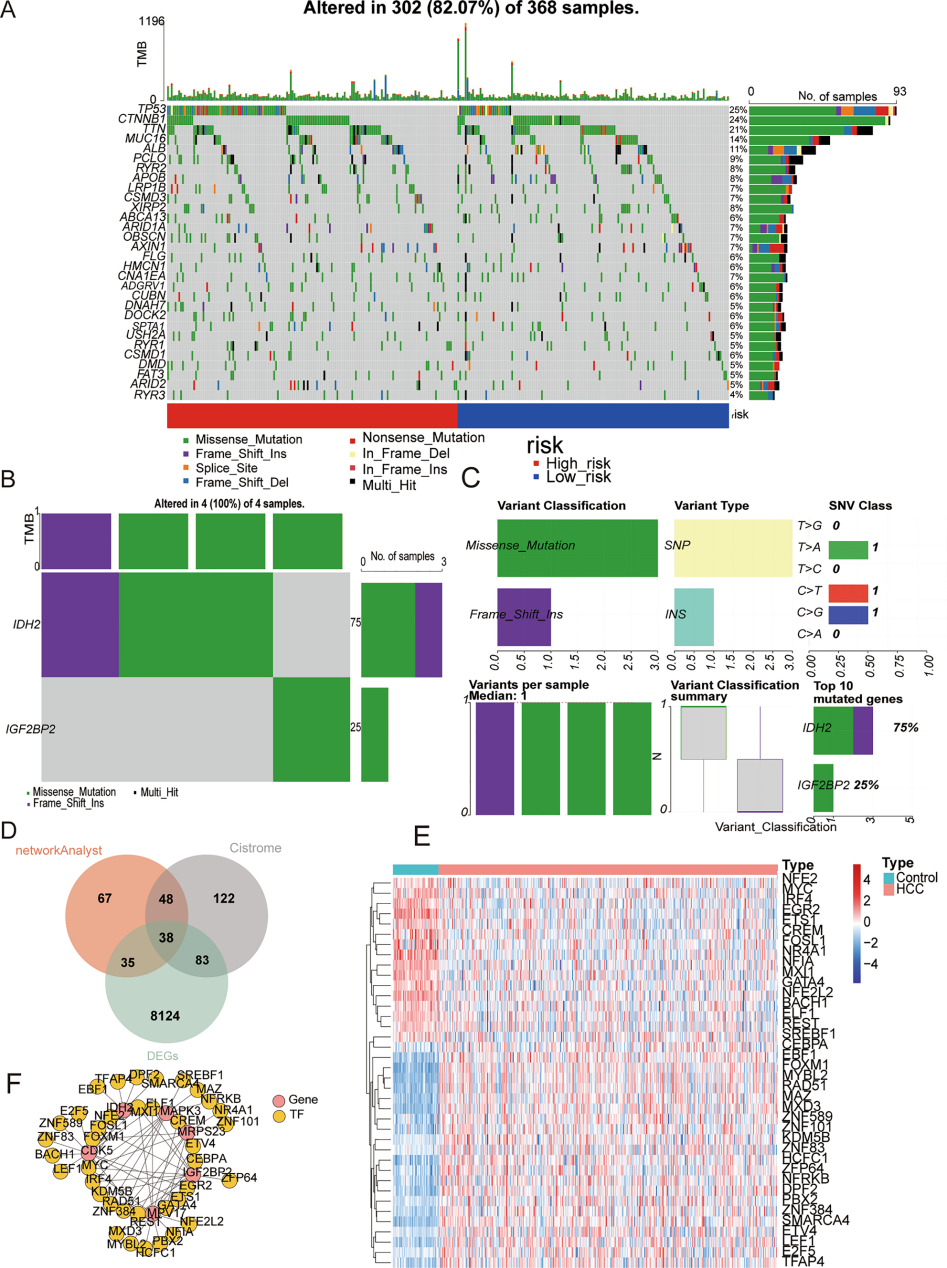
Mutation analysis and the construction of the transcription factors (TFs) regulatory network. In the higher risk categories, TP53 and CTNNB1 were more commonly mutated. **A** Mutation landscape analysis of high- and low-risk subgroups. Different types of mutations were shown in different colors. The blue represents the low-risk group sample, and the red represents the high-risk group sample. **B, C** Variation types of prognostic characteristic genes in the TCGA-HCC dataset. Only IDH2 and IGF2BP2 were mutated in few HCC samples. IDH2 was relevant to an single nucleotide polymorphism (SNP) mutation, and IGF2BP2 was relevant to an insulin gene (INS) mutation. **D** The TFs predicted by Cistrome database and Network Analyst were intersected with DEGs to obtain differentially expressed upstream TFs of OXPHOS-associated prognostic genes. **E** Heatmap of 38 TFs expression in TCGA-HCC dataset. **F** The regulatory network contained six OXPHOS-relevant prognostic genes and 38 TFs. Pink circles represented prognostic signature genes and yellow nodes represented TFs

To further investigate the distinction in mutations between two OXPHOS-relevant risk subgroups, we employed the ‘maftools’ package to visualise the distribution of the top30 mutated genes in HCC, revealing that TP53 and CTNNB1 were more frequently mutated in the higher risk groups (Fig. 7A). Furthermore, in order to investigate the mutation states associated with the six OXPHOS-relevant prognostic genes in HCC, we performed the corresponding analysis using the GSCALite. Only IDH2 and IGF2BP2 were mutated in few HCC samples (Fig. 7B, C). To pursue further exploration of the upstream regulatory mechanisms of OXPHOS-relevant prognostic genes, we proceeded with the fabrication of a TF regulatory network, where 38 differentitially expressed TFs common to the Cistrome database and NetworkAnalyst were represented targeting six OXPHOS-relevant prognostic genes (Fig. 7D–F), providing clues for future exploration of the regulation of the signature genes.

### The expression of OXPHOS-relevant prognostic genes in HCC

**Fig. 8:**
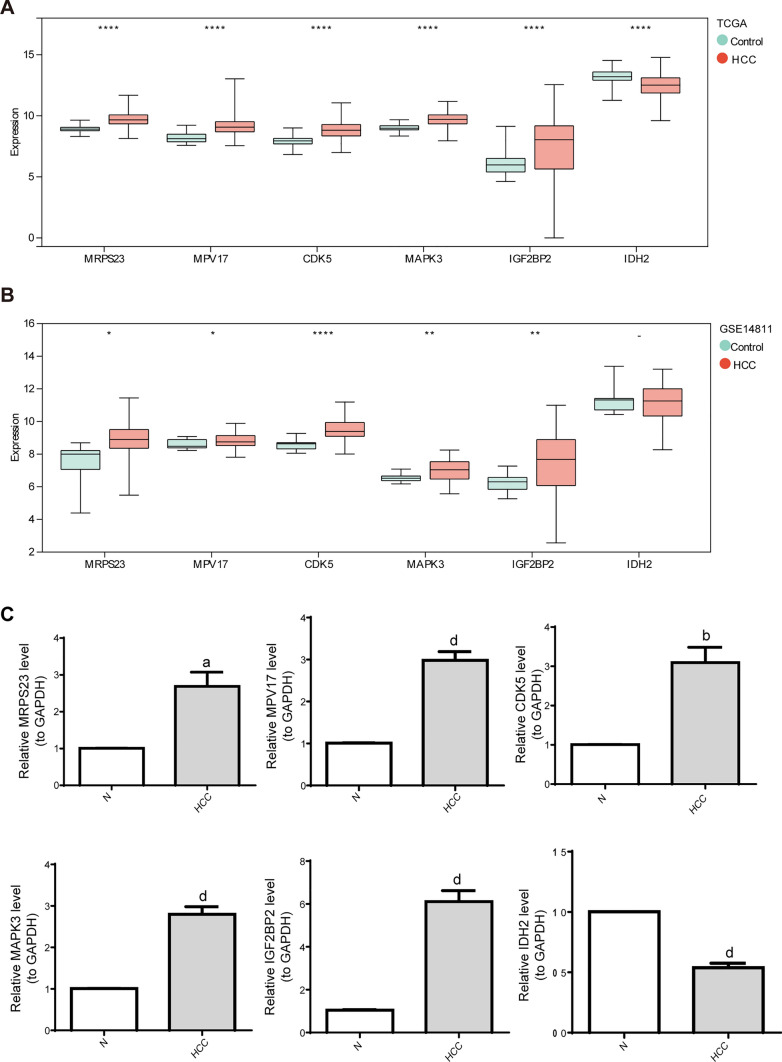
The expression verification of six prognostic characteristic genes. MRPS23, MPV17, CDK5, MAPK3 and IGF2BP2 expression was elevated in HCC patients. The box plots for the expression patterns of six genes in **A** TCGA dataset and (**B**) GSE14811 dataset. **C** Real Time Quantitative PCR (RT-qPCR) image was generated using Graphpad prism 6. ^a^P < 0.05, ^b^P < 0.01, ^c^P < 0.001, ^d^P < 0.0001

As illustrated in Fig. 8A, IDH2 was downregulated, and MRPS23, MPV17, CDK5, MAPK3, and IGF2BP2 presented elevated expression in HCC tissues in the TCGA-HCC cohort, otherwise, the same findings were appeared in GSE50579 except for IDH2 (Fig. 8B). For the RT-qPCR results, the expression of IDH2 was notably reduced, and MRPS23, MPV17, CDK5, MAPK3, and IGF2BP2 were markedly overexpressed in clinical HCC samples versus control samples, which were in accordance with the results in online datasets above (Fig. 8C).

## Discussion

Despite the current improvements in the treatment of HCC, the clinical outcome of patients is not promising and there is a lack of effective diagnostic and prognostic markers. Tumors are prone to metastasis and recurrence due to the inability to monitor the disease and provide effective treatment in a timely manner. Recently, OXPHOS has received extensive concern as an emerging target in antitumor therapy [9, 11, 26]. However, the OXPHOS-involved underlying mechanism and clinical utilization in HCC remain worth exploring.

In the study, we firstly identified 50 OXPHOS-related DEGs by differential expression analysis, most of which have been considerated within cancer progression. For example, SNHG3 which was involved in the miR-186-5p/ZEB1 pathway could mediate pathological invasion and metastasis as well as transforming breast cancer cells into epithelial-mesenchymal [27]. Furthermore, the functional annotation results of these DEGs enriched various processes that were reported in many cancers [28–31], including central carbon metabolism, Hepatitis B, ErbB signaling pathway, and TCA cycle.

We subsequently employed the TCGA-HCC, GSE76427, and ICGC-HCC datasets as the training and validation datasets and established the risk signature for HCC patients, which contained six OXPHOS-related genes (MRPS23, MPV17, MAPK3, IGF2BP2, CDK5, and IDH2). Most signature genes involved have been reported to be of importance in HCC development. CDK5 could functionate in hepatocellular tumorigenesis [32], and inhibition of CDK5 notably improved sorafenib resistance by interrupting treatment escape in HCC [33]. Down-regulation of IDH2 may induced HCC cell invasion inhibition via NF-x03BA;B signal [34], moreover, IDH2 expression was linked with survival as well as recurrence for HCC patients [35]. IGF2BP2, as an oncogene, enhanced FEN1 mRNA stability and maintained its expression by participating the N6-methyladenosine (m6A) modification to HCC cells [36]. In addition, although the MRPS23 influence on HCC remains unclear, there is evidence indicating that it can promote breast cancer metastasis by regulating OXPHOS with its arginine and lysine methylation, which has a significant reference value [37].

Furthermore, based on the OXPHOS-related signature constructed, we divided the whole data sets into high and low-risk subgroups. It is worth that patients with high-risk scores were accompanied with lower survival rates. At the meantime, the predictive accuracy of the signature was confirmed via the preferable AUC value. In addition, by combining the clinical features, we observed that the OXPHOS-related signature and pathologic T were the independent prognostic factors for HCC patients. The nomogram and calibration curves further affirmed the clinical significance of the prognostic signature.

In addition, the significant process for the low-risk populations were involved in the PPAR [38, 39], calcium [40], as well as insulin-related signaling pathways [41], and the high-risk group were spliceosome [42], ribosome [43], and cell cycle [44]. Most of them have been studied in cancers, guiding our follow-up research. On the other hand, the stromal score of the low-risk group was higher despite no significant differences between the immune score and ESTIMATE score observed in the two subgroups, that is, the immunotherapy might more benefit the populations with low-risk scores due to their higher immunogenic. Using the immune-related analyses of the signature, we found that among nine immune cells infiltration (B cells, CD8 T cells, cytotoxic cells, etc.) were notably higher in the low-risk group, which were contrary to the TH2 infiltration results.

It is evidenced that immune cell infiltration could linkage to survival of HCC patients [45]. The density of infiltrating tumor B cells and T cells, which are in intensive contact with each other and enhance local immune activation, correlates with the survival advantage of HCC patients [46]. Related studies have shown that NK cells and eosinophils exhibit strong tumor cytotoxic effects in the immune microenvironment [47, 48]. The imbalance of Th1 and Th2 cytokines in the microvasculature surrounding tumor tissue predicts the prognosis of HBV-related HCC and TACE surgery [49, 50]. Our results suggested that patients in the high and low risk groups had significant differences in tumour cell infiltration including B cells, CD8 T cells, cytotoxic cells, DC, eosinophils, neutrophils, NK cells, Tcm, Th17 cells and Th2 cells. Those immune cell members were of primal importance for HCC, in turn demonstrating that our constructed prognostic signature may be correlated with tumor immune infiltration, affecting the prognosis of patients.

Using the susceptibility analysis of patients in the different risk groups to immune checkpoint and immunotherapy, it is suggested that the low-risk group had higher PD-L1 expression, and the TIDE score and T-cell exclusion score were lower in patients with low-risk scores. Accumulating evidence indicated that monoclonal antibodies targeting PD-1 are immunotherapeutic agents for multiple malignancies, and PD-L1 is also classified as a second-line agent for HCC patients [51, 52]. Tumor sensitivity to drugs is frequently associated with the therapeutic efficacy of drugs and affects patient survival prognosis. A previous study suggested that the anti-tumor immunity effects of CDK5 inhibition might be achieved by mediating the continuous expression of PD-L1 transcription inhibitors [53]. Another study of oral squamous cell carcinoma suggested that IGF2BP2 regulates immune inflammation cytokines and correlates with immune infiltrates [54, 55]. Likewise, HCC patients with low-risk scores had better survival and outcomes for immunotherapy in the current study. For the chemotherapy drugs associated with different risk groups, it is shown that lapatinib and paclitaxel might benefit the high-risk population, while the low-risk population could profit from more multiple antineoplastic drugs such as oxaliplatin, ruxolitinib.

Compared to the prognostic analysis of individual genes, the results of a collective signature are more broadly adapted and comprehensively reflect changes across the entire genome, providing more accurate biological information. However, there are still some deficiencies in our study. First of all, the findings need to be further evidenced through more basic experiments or clinical studies. Second, the signature genes-related process and biological significance remains to be penetrated in further by experimental mechanism exploration and clinical application research. These questions will be paid continuous attention in further researches. In general, the clinical utilize of the OXPHOS-relevant gene signature was evaluated using the construction of the nomogram. The correlation of immune cell infiltration and risk score as well as the response to immunotherapy and chemotherapy for HCC cohorts were investigated between the two risk groups. Finally, the gene expression, mutation states, and the TF regulatory network of the signature genes were preliminary explored in the current study.

## Conclusion

In conclusion, an OXPHOS-related signature with appreciable accuracy for survival prediction of HCC patients was firstly identified in our study. It could improve survival in HCC patients by reasonably screening out patients who may be effective in immunotherapy, chemotherapy, and targeted therapy. However, there is still a long way to go in the clinical application stage. In the future, we will continue to focus on and explore ways to compensate for the limitations of our results.

## Data Availability

TCGA-HCC dataset was downloaded from the TCGA (https://portal.gdc.cancer.gov/) database. GSE74627 and GSE50579 datasets were soured from the GEO (https://www.ncbi.nlm.nih.gov/geo/) database. ICGC-HCC dataset was derived from the ICGC (https://docs.icgc.org/) database**.**

## Abbreviations

LASSO: Least absolute shrinkage and selection operator
ROC: Receiver operating characteristic
LC: Liver cancer
HCC: Hepatocellular carcinoma
ISG15: Interferon-stimulated gene 15
OXPHOS: Oxidative phosphorylation
TCGA: The Cancer Genome Atlas
GEO: Gene Expression Omnibus
ICGC: International Cancer Genome Consortium
DEGs: Differentially expressed genes
GO: Gene Ontology
KEGG: Kyoto Encyclopedia of Genes and Genomes
CC: Cellular component
MF: Molecular function
BP: Biological process
LASSO: Least absolute shrinkage and selection operator
GSEA: Gene Set Enrichment Analysis
NES: Normalized enrichment scores
TME: Tumor microenvironment
ICB: Immune checkpoint blockade
TIDE: Immune Dysfunction and Exclusion
IC50: Half-limiting dose
TF: Transcription factor
SNP: Single nucleotide polymorphism
INS: Insulin gene
